# The complete mitochondrial genome of *Halocosa hatanensis* (Araneae: Lycosidae)

**DOI:** 10.1080/23802359.2020.1806751

**Published:** 2020-08-17

**Authors:** Jing-Yi Chen, Run-Biao Wu, Zhi-Sheng Zhang

**Affiliations:** Key Laboratory of Eco-environments in Three Gorges Reservoir Region (Ministry of Education), School of Life Sciences, Southwest University, Chongqing, China

**Keywords:** *Halocosa hatanensis*, mitochondrial genome, phylogenetic analysis

## Abstract

The complete mitochondrial genome sequence of a desert wolf spider, *Halocosa hatanensis* (Lycosidae) was reported. The circular molecule is 14,257 base pairs (bp) in length and consisted of 77.8% AT nucleotides, containing two ribosomal RNA genes, 22 transfer RNAs, 13 protein-coding genes (PCGs), 1 control region, and 1 AT-rich region. The length of 13 PCGs is 10,782 bp in total, encoding 3638 amino acids. The phylogenetic analyses upon mitogenomes proves its relatively basal position within Lycosidae. It would provide further evolutionary research data for the wolf spider family Lycosidae.

The wolf spider family Lycosidae is a great diverse group with 125 genera and 2431 species worldwide (World Spider Catalog 2020). But, the mitogenomes of only four Lycosidae species were sequenced, including *Pardosa laura* (NC025223), *Pirata subpiraticus* (NC025523), *Wadicosa fidelis* (NC026123) and *Arctosa tanakai* (CNA0013709). Here, the mitogenome of one more Lycosidae species, *Halocosa hatanensis*, was sequenced and reported.

The species, *Halocosa hatanensis* (Urita et al. 1993) is one of the three species of the wolf spider genus *Halocosa* (Azarkina and Trilikauskas, 2019), which lives in the saline habitats of Eurasia (World Spider Catalog 2020). Our survey shows that *H. hatanensis* is widely distributed in Northwest China, including South Xinjiang, Qinghai, Inner Mongolia and Ningxia (unpublic data), as a representative of spiders adapted the arid and desert area. It might play an important role in the control of pests of arid habitats.

Samples were collected at the side of Taitema Lake (N39°30.242′, E88°18.912′, Alt. 845 m) in Ruoqiang County, Xinjiang Uygur Autonomous Region of China on June 2014. After morphological identification, the specimens (sample number: XJRQ-14-31) were stored at −70 °C in the Arachnological collection (School of Life Sciences, Southwest University, Chongqing, China, SWUC). Genomic DNA was extracted from tissue using a DNeasy Blood and Tissue Kit (Qiagen, Valencia, CA, USA). DNA concentration was quantified using Qubit 2.0 (Invitrogen, Life technologies). We sequenced the complete mitochondrial genome of *H. hatanensis* using Illumina HiSeq X Ten platform with the insert-size 300 bp paired-end (PE) at 2 Gb depth at BGI-Wuhan, China. Raw sequencing data were assembled and annotated using mitoZ (Meng et al. 2019). The assembled data is now uploaded to the NCBI database and the GenBank accession number is MT174468.

The complete mitochondrial genome of *H. hatanensis* is 14,257 bp in length, which consist 37 genes, including 13 protein-coding genes (PCGs), 22 tRNAs, and 2 rRNAs. The overall base composition of the genome is 43.3% A, 14.1% C, 8.1% G, and 34.5% T(U), exhibiting an obvious A + T bias (77.8%). The AT-skew (0.113) for the whole mitogenome is slightly positive while GC-skew (–0.270) is negative, indicating a higher occurrence of As than Ts and Cs than Gs. Among 13 PCGs, five (COX1, ATP8, CYTB, ND1, ND2) started with ATT, four (ATP6, ND3, ND4, ND6) started with ATA, two (COX2, COX3) started with TTG, one (ND5) started with TTA and one (ND4L) started with AAT. Nine genes ended with TAA as stop codon, two genes (ND4, ND4L) had incomplete stop codons, one (ND5) ended with TAT, and one (ND1) ended with AAT.

This mitochondrial genome was analyzed, along with four previously reported lycosid species’ mitogenomes and another species of Pisauridae, *Dolomedes angustivirgatus* (NC031355) downloaded from GenBank. 13 PCGs were extracted and concatenated using PhyloSuite v1.2.1 (Zhang et al. 2020). The best partitioning scheme and evolutionary models were selected using PartitionFinder2 (Lanfear et al. 2016). We conducted a Bayesian Inference (BI) tree using PhyloSuite to analyze the phylogenetic relationship within Lycosidae (Figure 1). The phylogenetic tree shows the monophyly of Lycosidae and the basal infrafamilial position of *H. hatanensis.*

**Figure 1.**
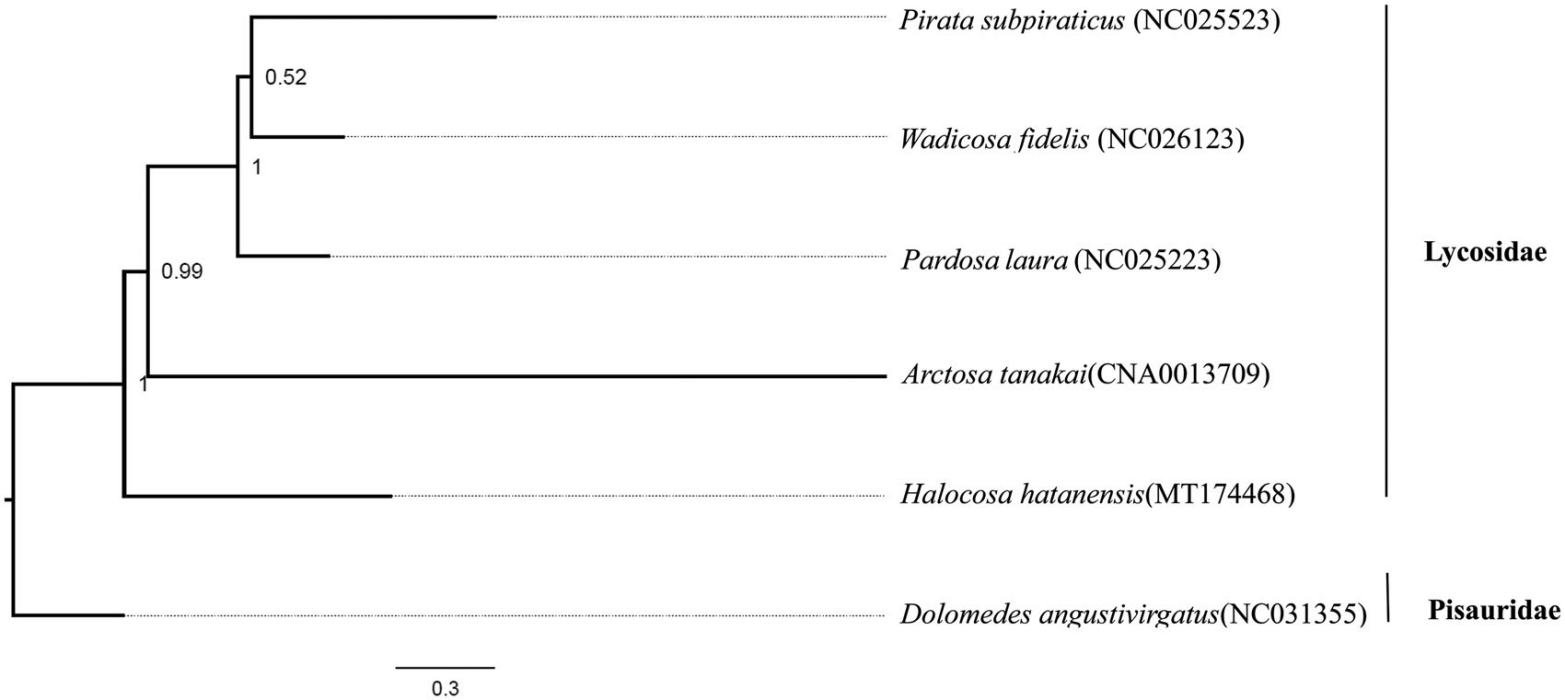
Phylogenetic relationships between *Halocosa hatanensis* and other Lycosidae species (GenBank accession numbers provided) inferred from 13 PCGs. Numbers at nodes indicate posterior probability (PP) values.

## Data Availability

The mitochondrial sequence of *Pardosa laura*, *Pirata subpiraticus* and *Wadicosa fidelis* are openly available in NCBI at https://www.ncbi.nlm.nih.gov/nucleotide/, accession number (NC025223, NC025523, NC026123). The raw sequence for *Halocosa hatanensis* has been deposited in the NCBI SRA: BioProject ID PRJNA608599 at https://www.ncbi.nlm.nih.gov/sra/SRR11196370, accession number (MT174468).The mitochondrial sequence of *Arctosa tanakai* is openly available in CNGBdb at https://db.cngb.org/search/assembly/CNA0013709/, accession number (CNA0013709).

## References

[CIT0001] Azarkina GN, Trilikauskas LA. 2019. *Halocosa* gen. n., a new genus of Lycosidae (Araneae) from the Palaearctic, with a redescription of *H. cereipes* (L. Koch, 1878). Zootaxa. 4629(4):555–570. 10.11646/zootaxa.4629.4.4 31712501

[CIT0002] Lanfear R, Frandsen PB, Wright AM, Senfeld T, Calcott B. 2016. PartitionFinder 2: new methods for selecting partitioned models of evolution for molecular and morphological phylogenetic analyses. Mol Biol E. 34(3):772–773. 10.1093/molbev/msw260 28013191

[CIT0003] Meng GL, Li YY, Yang CT, Liu SL. 2019. MitoZ: a toolkit for animal mitochondrial genome assembly, annotation and visualization. Nucleic Acids Res. 47(11):e63. 30864657 10.1093/nar/gkz173 PMC6582343

[CIT0004] Urita , Tang GM, Song DX. 1993. Two new species of the genus *Pardosa* from Inner Mongolia, China (Araneae, Lycosidae). J Inner Mongolia Normal Univ (Nat Sci Ed). 3:46–49.

[CIT0005] World Spider Catalog. 2020. World spider catalog. Version 21.0. Natural History Museum Bern; [accessed 2020 Jun 13]. https://wsc.nmbe.ch/

[CIT0006] Zhang D, Gao FL, Jakovlić I, Zou H, Zhang J, Li WX, Wang GT. 2020. PhyloSuite: an integrated and scalable desktop platform for streamlined molecular sequence data management and evolutionary phylogenetics studies. Mol Ecol Resour. 20(1):348–355. 31599058 10.1111/1755-0998.13096

